# Arogya Kiran model for early detection of diabetes and hypertension: an initiative for the community and by the community in India

**DOI:** 10.1186/1472-6963-14-S2-P113

**Published:** 2014-07-07

**Authors:** Ruchi Sogarwal, Sunil Mehra

**Affiliations:** 1 MAMTA Health Institute for Mother and Child, New Delhi, Delhi, India

## Background

The growing scale of diabetes and hypertension in India, calls for a strong policy focus on early detection, prevention and management of these diseases. Recognizing the fact, recently Government of India has given strong emphasis on early detection, as one of the important strategies in tackling the burden of diabetes and hypertension. With the support of Bristol-Myers Squibb Foundation, MAMTA Health Institute for Mother and Child introduced a strategic prevention initiative for identification of undiagnosed cases in selected districts of India. The purpose of this article is to describe this initiative named as ‘Arogya Kiran (A ray of hope for health)’ model and its impact on early detection of diabetes and hypertension.

## Methods

An Arogya Kiran model is a strategic initiative for the community and by the community, which is conceptually based on combination of socio-ecological and patient empowerment models that consider the complex interplay between individual, community and environmental factors. A non-randomised, controlled, before-after quasi-experimental trial was conducted in intervention and comparison area to assess the impact of the initiative.

## Results

600 community volunteers (i.e. 400 rural, 200 urban) and 200 school teachers were identified and trained in prevention and management of diabetes and hypertension, and are named as Arogya Kiran. During Aug 2013 to Feb 2014, 600,000 adult men and women of both rural and urban areas; 20,000 adolescent students (from 100 schools) and; 3,000 employees from 30 workplaces were covered. As compared to baseline, 1.36% relative increase has been contributed by Arogya Kiran in identifying “at-risk’ individuals for diabetes and hypertension, and in connecting to healthcare providers for final diagnosis and treatment. Interestingly, the average cost of introducing this model is USD 98.7 per member per year, which includes training for three days (USD 25.8 per member); operational cost (USD 3.3 per month per member); and quarterly follow-up meeting cost (USD 8.2 per month per member).

## Conclusion

The Arogya Kiran model has shown pathway and understanding to move towards a comprehensive, coordinated, and efficient community based program, a step forward to realize the vision of a fully integrated system for patients, families, and clinicians across the continuum of care. Case detection of undiagnosed diabetes and hypertension through this model is low but definitive cost-effectiveness calculations must be done before advocating for scale-up.

